# Supplementary material for: Loss of Cnot6l Impairs Inosine RNA Modifications in Mouse Oocytes
Source: Int J Mol Sci. 2021 Jan 26;22(3):1191. doi: 10.3390/ijms22031191 (PMC7865253; doi:10.3390/ijms22031191)
Supplement: Supplementary file 1 [file ijms-22-01191-s001.zip › Supplemental Files/Supp_S3_new.pdf]

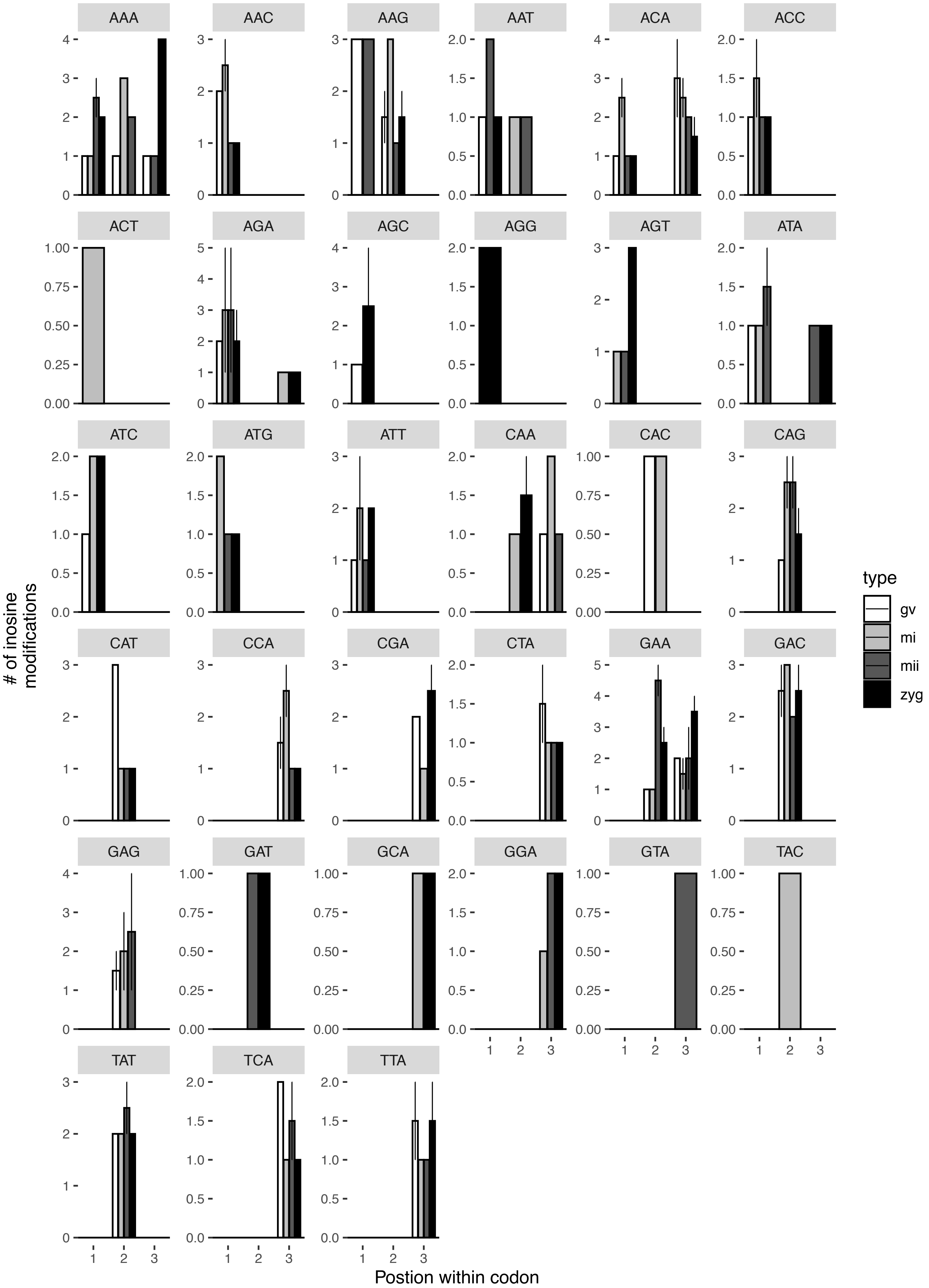

Supplemental Figure S3. The number of inosine RNA modifications within mRNA codons of Cnot6l-/- oocytes, eggs, and zygotes within the total mRNA. Each codon position is denoted (1-3) to quantify the number of detected inosine mRNA modifications.
