# Supplementary material for: Loss of Cnot6l Impairs Inosine RNA Modifications in Mouse Oocytes
Source: Int J Mol Sci. 2021 Jan 26;22(3):1191. doi: 10.3390/ijms22031191 (PMC7865253; doi:10.3390/ijms22031191)
Supplement: Supplementary file 1 [file ijms-22-01191-s001.zip › Supplemental Files/Supp_S7_new.pdf]

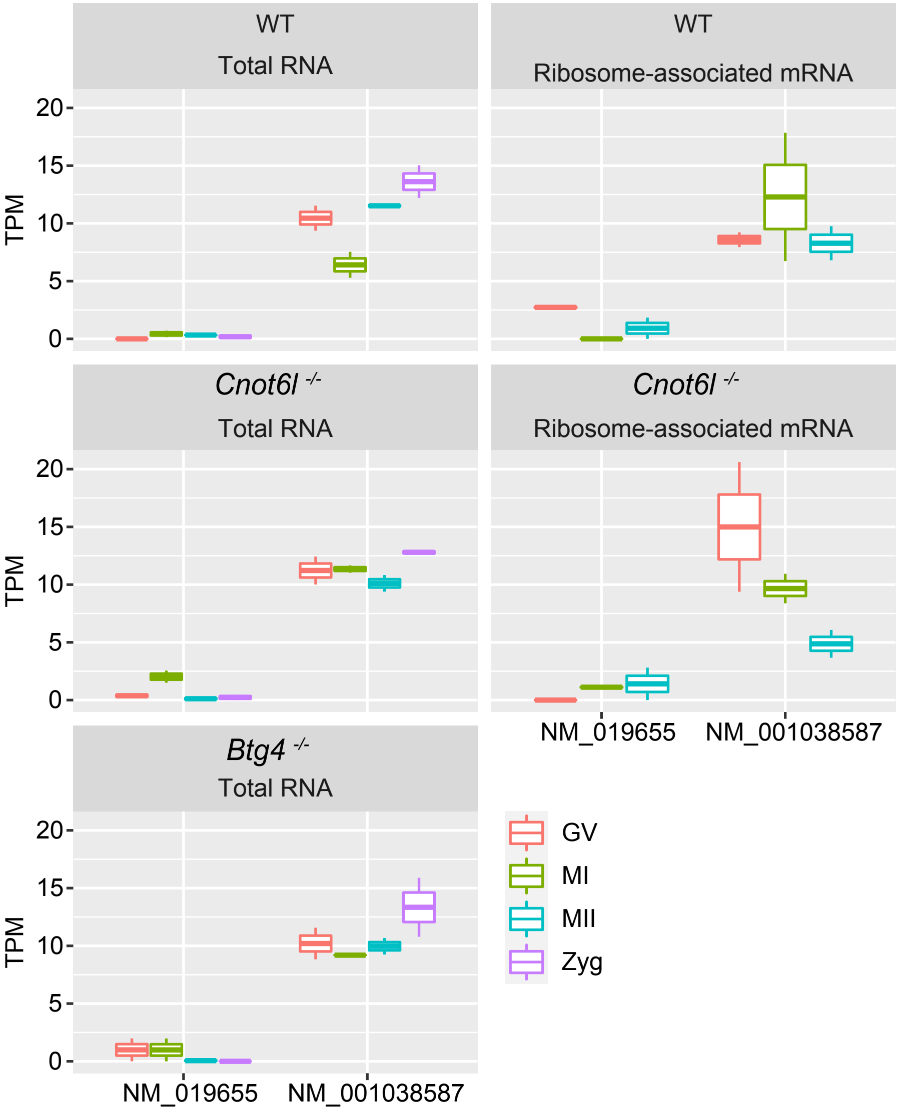

Supplemental Figure S7. Adar isoform abundance. Adar isoform abundance in total RNA and ribosome-associated mRNA from WT, Cnot6l-/-, and Btg4-/- oocytes and zygotes. TPM= transcripts per million.
