# Supplementary figures and images for: Loss of Cnot6l Impairs Inosine RNA Modifications in Mouse Oocytes
Source: Int J Mol Sci. 2021 Jan 26;22(3):1191. doi: 10.3390/ijms22031191 (PMC7865253; doi:10.3390/ijms22031191)

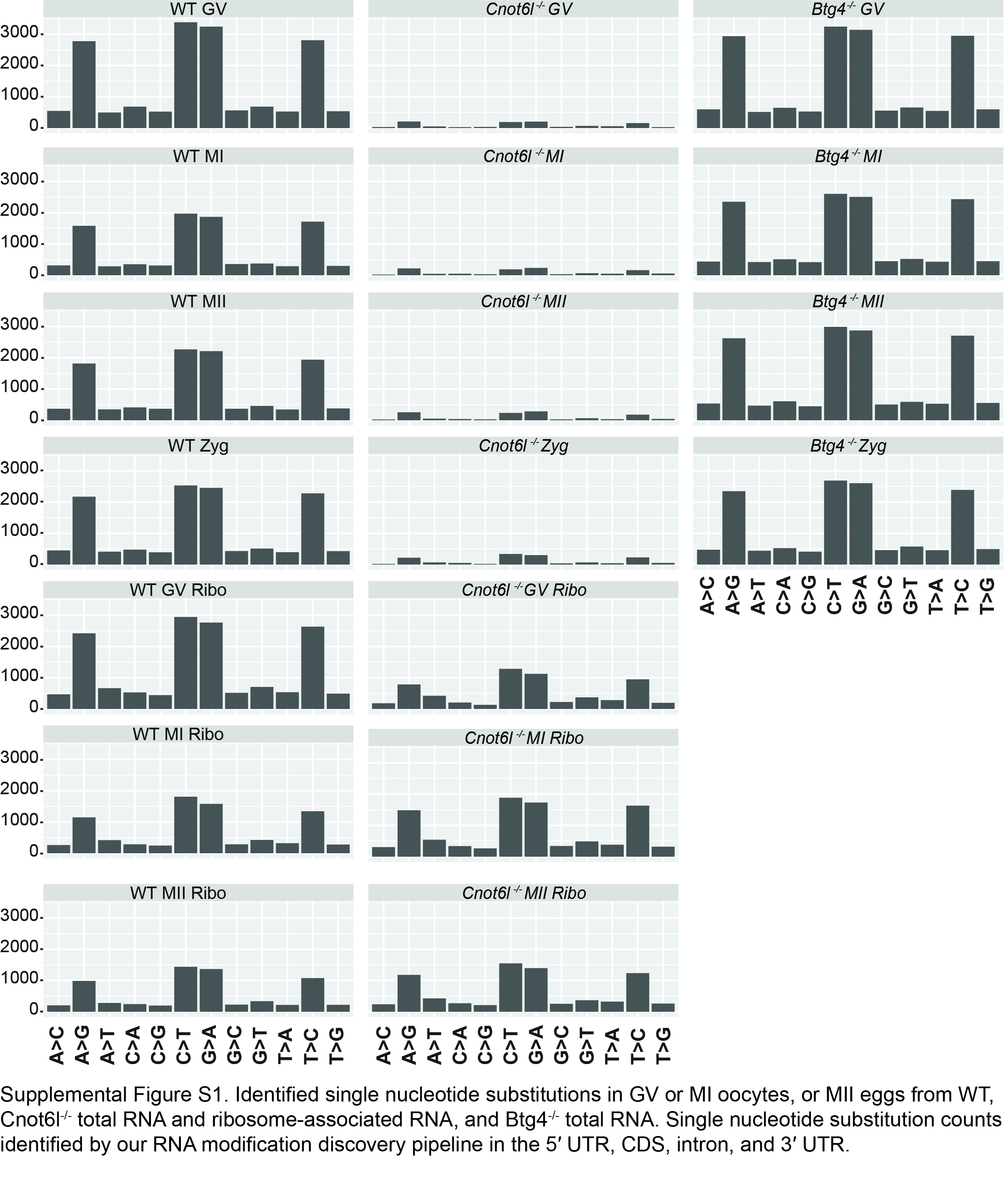

Supplement: Supplementary file 1 [file ijms-22-01191-s001.zip › Supplemental Files/Supp_S1_new.jpg]

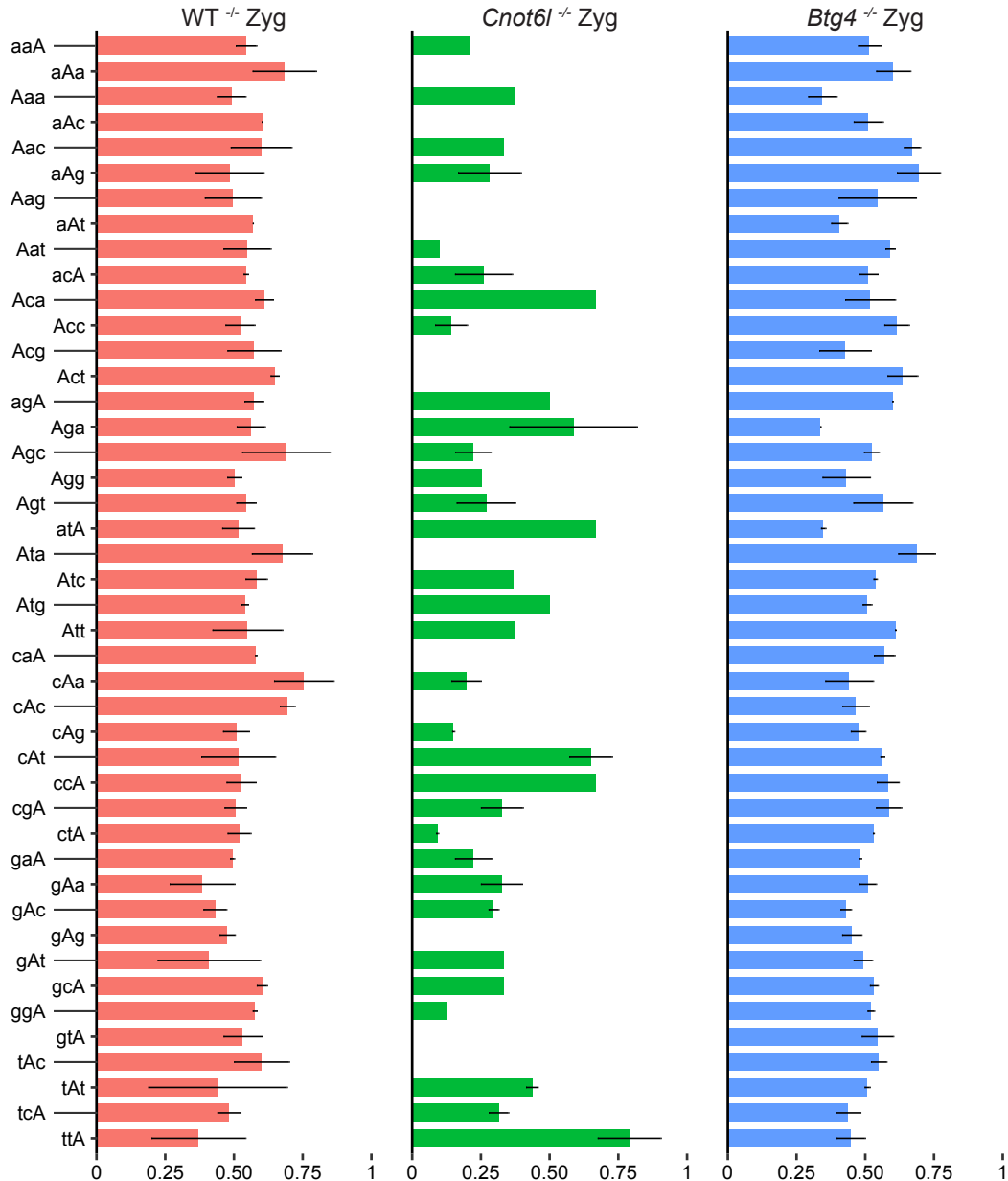

Supplement: Supplementary file 1 [file ijms-22-01191-s001.zip › Supplemental Files/Supp_S6_new.pdf]
